# Sources of potential bias when combining routine data linkage and a national survey of secondary school-aged children: a record linkage study

**DOI:** 10.1186/s12874-020-01064-1

**Published:** 2020-07-02

**Authors:** Kelly Morgan, Nicholas Page, Rachel Brown, Sara Long, Gillian Hewitt, Marcos Del Pozo-Banos, Ann John, Simon Murphy, Graham Moore

**Affiliations:** 1 Centre for the Development and Evaluation of Complex Interventions for Public Health Improvement (DECIPHer), School of Social Sciences, Cardiff University, Cardiff, CF10 3BD UK; 2 Swansea University Medical School, Swansea University, Swansea, UK

**Keywords:** Data linkage, Bias, Consent, Success rate, Adolescents, School, Wales

## Abstract

**Background:**

Linking survey data to administrative records requires informed participant consent. When linkage includes child data, this includes parental and child consent. Little is known of the potential impacts of introducing consent to data linkage on response rates and biases in school-based surveys. This paper assessed: i) the impact on overall parental consent rates and sample representativeness when consent for linkage was introduced and ii) the quality of identifiable data provided to facilitate linkage.

**Methods:**

Including an option for data linkage was piloted in a sub-sample of schools participating in the Student Health and Wellbeing survey, a national survey of adolescents in Wales, UK. Schools agreeing to participate were randomized 2:1 to receive versus not receive the data linkage question. Survey responses from consenting students were anonymised and linked to routine datasets (e.g. general practice, inpatient, and outpatient records). Parental withdrawal rates were calculated for linkage and non-linkage samples. Multilevel logistic regression models were used to compare characteristics between: i) consenters and non-consenters; ii) successfully and unsuccessfully linked students; and iii) the linked cohort and peers within the general population, with additional comparisons of mental health diagnoses and health service contacts.

**Results:**

The sub-sample comprised 64 eligible schools (out of 193), with data linkage piloted in 39. Parental consent was comparable across linkage and non-linkage schools. 48.7% (*n* = 9232) of students consented to data linkage. Modelling showed these students were more likely to be younger, more affluent, have higher positive mental wellbeing, and report fewer risk-related behaviours compared to non-consenters. Overall, 69.8% of consenting students were successfully linked, with higher rates of success among younger students. The linked cohort had lower rates of mental health diagnoses (5.8% vs. 8.8%) and specialist contacts (5.2% vs. 7.7%) than general population peers.

**Conclusions:**

Introducing data linkage within a national survey of adolescents had no impact on study completion rates. However, students consenting to data linkage, and those successfully linked, differed from non-consenting students on several key characteristics, raising questions concerning the representativeness of linked cohorts. Further research is needed to better understand decision-making processes around providing consent to data linkage in adolescent populations.

## Background

Data linkage refers to the “process of pairing records from two files and trying to select the pairs that belong to the same entity” [1]. Linking routinely collected electronic data such as health and other administrative records has garnered increasing interest among policy makers, clinicians and researchers given that it can represent a rapid and relatively inexpensive approach to studying the aetiology of disease and injury, and wider (e.g. socio-ecological) determinants of health. Data linkage can inform evidence-based policymaking cycles, by i) generating epidemiological evidence with a high level of external validity; and ii) facilitating the evaluation of population level polices and targeted interventions [2, 3].

A benefit of using administrative data for research purposes is potential access to population-level data with a high-level of granularity, for instance, data available at an individual or household level. That said, potential drawbacks of using linked administrative data are also well established, specifically those pertaining to data completeness (or lack thereof) and quality [4]. Incomplete data could occur in the traditional sense, i.e. due to missing observations, or because an individual has not had any prior interaction with a particular service, for example, no medical exam report [5]. Alternatively, missing data could result from inaccurate linkage, whereby an individual’s data could not be linked to routine records. Further challenges of data linkage relate to the quality of data provided, with study outcomes often limited by the types of data collated. In cases where health professionals will record incidents of patients attending appointments and events such as mental health diagnoses, indicators represent a combined measure of help-seeking, recognition, recording and incidence, meaning that health related problems experienced by those who do not access services are not captured [6]. Similarly, when studying the impacts of alcohol use, for example, routine data may only capture more serious incidences, for instance where use has become sufficiently severe enough to require a hospital admittance.

Combining routine data with self-report survey data can help offset the respective limitations of each data type [2]. Indeed, the benefits of linking individual-level health records with data collected for a study are well-characterised and include a reduction in the reliance on self-report data, a reduced burden upon participants in comparison with other longitudinal study designs, and the ability to address issues of missing data [3]. In recent years, a large number of UK-based longitudinal studies have linked participant survey study datasets to administrative records in so-called ‘hybrid cohorts’ [7]. However, linking such datasets can pose a unique set of methodological challenges. For instance, the process of data linkage requires the provision of person-identifiable data, usually name, address, sex, date of birth and, ideally, a unique national identification number (e.g. National Health Service number or a pupil’s UPN (unique pupil number) in the UK) [8]. However, while data linkage is relatively straightforward in cases where a national identification number is provided, yielding good linkage outcomes [8], linkage based on alternative identifiers (i.e. “fuzzy matching” of name, address and date of birth) has tended to yield higher rates of linkage error, commonly due to inaccurate or incomplete provision of identifiable data. Therefore, the ability to accurately collect identifiable information during large scale surveys (i.e. the logistical constraints coupled with a participant’s ability to accurately recall different types of identifiable information), alongside the potential effect of linkage error on study outcome measures, [9] are important considerations within data linkage study designs. For example, it is important to consider whether the age of a young person impacts upon the precision with which they can provide postcode data.

To mitigate any risks to participant anonymity and appease privacy concerns, steps are taken to ensure datasets are pseudonymised prior to linkage [10]. During this process data are anonymised for the people who receive it (e.g. a research team) but codes are retained which would enable others, for instance those responsible for the individual’s care, to identify an individual from it [11]. Previous research has indicated that public willingness to share patient data is often contingent upon a number of concerns including data de-identification and privacy, trustworthiness of those accessing data, transparency of data use, security controls and the ability to retain control over future use [12–14]. As such, best practice principles are known to include independent governance committees to oversee processes, public engagement to fully realise the public benefits of data-based health research, and the use of dynamic consent approaches [12].

Obtaining informed consent is usually a pre-requisite for linking participant study data with routine data in many countries, including the UK. That said, a study exploring consent preferences among adults revealed that most found it appropriate to conduct data linkage projects without consent, predicated on the condition that tasks are separated in a way that prevents researchers from receiving identifiable data [12]. A major concern surrounding consent is the issue of non-consent bias. This may occur if some individuals are less likely to consent than others, and that this non-consent is systematically related to characteristics of the respondent [13]. In addition, low consent rates may increase the variance of statistical estimates by limiting the size of the sample available for analysis [14]. For instance, studies of rare events, or rare events such as suicide, may be particularly vulnerable to selection bias because the number of available samples and data are inherently low for each condition [15].

A qualitative investigation into young people’s views on data linkage in the UK by Audrey and colleagues [16], for example, found that 17–19 year olds weighed up the sensitivity of personal data and the anticipated benefits of the research before deciding whether to provide consent for linkage. The process of obtaining consent takes on an added level of complexity when the study population concerns children. Informed parental/legal guardian consent is often a key requirement in order to protect young people from any potential harms or risks they may be exposed to via participation in empirical research, and hence important consideration should be given to the type of parental consent processes adopted. Active consent procedures (so-called ‘opt-in’ consent) require parents to return a signed consent form indicating whether they wish their child to participate in the research. In contrast, passive consent procedures (known as ‘opt-out’ consent) require parents to respond only if they do not wish their child to participate; with non-response taken to indicate parental assent. Schools often adopt passive approaches when conducting general health research based on convenience and topics of low sensitivity [17].

Whilst it is important that participants are fully informed and free to make decisions, maximising recruitment is an essential step towards ensuring representativeness within a study population, and lower participation rates and socio-demographic biases have been associated with parental opt-in procedures [18–20]. For example, meta-analysis by Liu et al., [20] revealed significantly lower consent rates among studies requiring active- compared with passive parental consent. Among studies reviewed, response rates ranged from 29 to 60% for studies adopting active consent procedures [21–24] and 79–100% among those in which passive consent was preferred [17, 25, 26]. The use of active consent was also shown to lead to systematic sample bias due to misrepresentation of certain populations [20]. Specifically, high-risk youths, males and older children were underrepresented among studies using active consent, leading the authors to highlight a need for balance between protecting young people on the one hand and ensuring inclusivity on the other. Consequently, whilst active parental consent offers the most conservative approach, concerns remain regarding the exclusion of the voices of high-risk groups of young people from research, and the prospective implications this may have for the external validity of findings drawn from such studies and the likelihood of basing policy decisions on data which exclude the most at risk groups [27].

Given the richness of secondary data available to researchers, and the growing international infrastructure dedicated to facilitating data linkage [3, 28], there is an increasing need to better understand who is (or is not) providing consent and to explore the impact (if any) that requesting consent to link data may have on study completion rates and sample representativeness.

The current study had three principal aims: i) to explore the impact of introducing routine data linkage on rates of parental opt-out within a national survey of secondary school-aged children; ii) to examine the characteristics of young people who provide consent for data linkage compared to those who do not; and iii) to ascertain the quality of identifiable data provided by the young people consenting to linkage.

## Methods

### Study design and participants

Data were collected through the 2017 School Health Research Network (SHRN) Student Health and Wellbeing (SHW) Survey, of which 193 schools (out of a possible 212) participated. The survey is a cross-sectional, school-based survey that explores a range of adolescent health behaviours, including substance use, physical activity, and mental wellbeing. It is administered to 11–16 year olds (although where schools have a sixth form, these students are also included if the school wishes) biennially between September and December via an electronic self-completion survey. Consent to participate in the survey is required at three levels; school, parent, and student. School level consent was first obtained from schools who wished to participate; parents/guardians were then informed about the survey via two standard school communication channels (i.e. letter, email or text message), where they were given the opportunity to withdraw their child via a process of ‘opt-out’ consent; and student consent was obtained at the beginning of the survey. Surveys were available in both English or Welsh language and data collection took place within the classroom environment. Participants were assured of anonymity and confidentiality prior to completion.

### Data linkage pilot

**Fig. 1:**
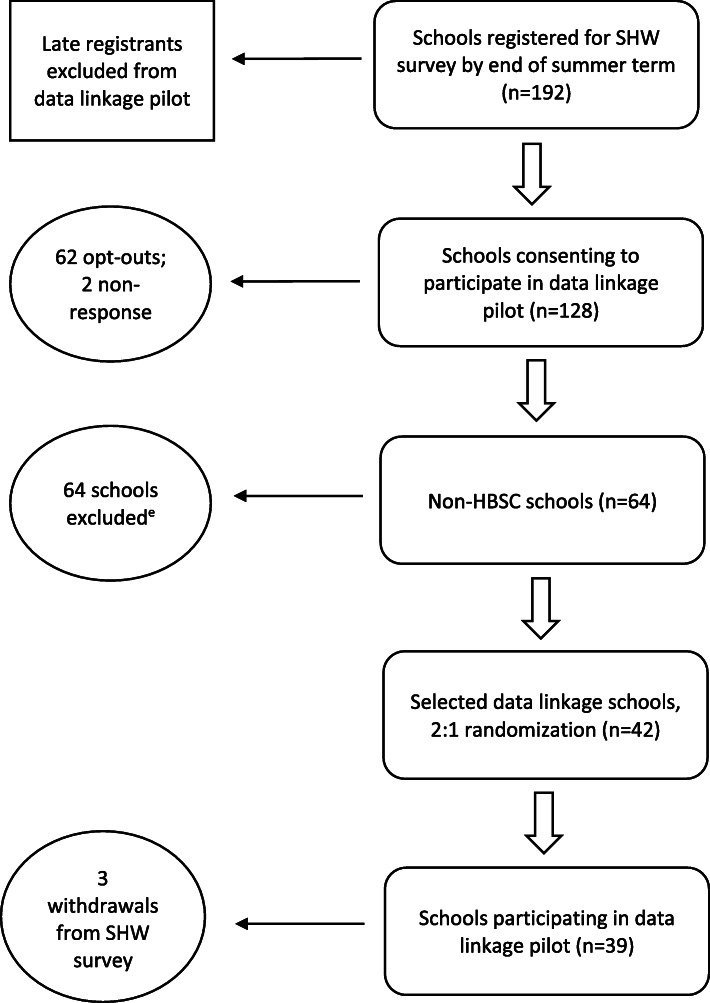
Flow chart of school recruitment process for data linkage pilot study. This included 63 HBSC schools and 1 non-HBSC school. The decision to omit the non-HBSC school from randomisation was taken by the researchers as the school was considered not to have had adequate time to make an informed decision regarding participating in the pilot due to it registering so close to the initial deadline

An embedded pilot design was employed in the 2017 survey, whereby a random sample of participating schools (*n* = 42) took part in an enhanced survey which sought to collect personal identifiers from students (i.e. first name, surname, date of birth and residential postcode) to facilitate longitudinal analysis and linkage to routinely collected data (see Fig. 1 for flow diagram of recruitment process). Since 2017, the World Health Organisation’s Health Behaviours in School-aged Children (HBSC) survey has been nested within the SHW survey and a sub-sample of SHRN schools complete the HBSC questionnaire (‘HBSC schools’). In order to ensure that the international HBSC survey response rates were not affected by the additional consent procedures for data linkage, only non-HBSC schools were considered for inclusion in the study. In total, 39 of the 42 schools participated, after three withdrew from the survey completely. Whilst pilot schools received the same questionnaire as non-pilot schools, in the two-week period preceding the survey, students within each pilot school were shown a video outlining survey details, the reasons for collecting unique identifiers, how data would be used, including the process of data linkage, and procedures for ensuring anonymity. These themes were revisited at the end of the questionnaire and prior to consent being sought for linkage. Schools were instructed to give a leaflet to students who missed the video or arrange an additional viewing.

After the main part of the questionnaire, students saw a page that reiterated why they were being asked for identifying information, that their consent was needed for longitudinal and data linkage research and that providing identifying information was optional. There followed separate consent statements for longitudinal and data linkage research (i.e. “I give my permission for researchers to use my survey answers for longitudinal research” and “I give my permission for researchers to use my survey answers for data linkage research”) for which students selected either ‘Yes’ or ‘No’. If ‘Yes’ was selected for either statement, boxes appeared on screen for students to complete their name, date of birth and postcode (two alphanumeric codes typically comprising seven digits). Students entered information manually for date of birth and postcode, rather than these being drop down lists, but the number of digits that could be entered was restricted to viable limits. Students were advised to give the postcode of the house where they lived most of the time, if they lived in more than one place.

### Measures

The wider SHW survey draws together questions from a range of sources. Questions reported here on demographics and substance use were drawn from the HBSC survey and questions on truancy and exclusions were developed for the survey and are detailed in full below.

#### Socio-demographic characteristics

Students were asked to report their gender (boy/girl) and school year (a proxy for age). Family Affluence Scale (FAS) was derived from six items which asked; 1) “Do you have your own bedroom?” 2) “How many computers does your family own?” 3) “Does your family own a car, van or truck?” 4) “Does your family have a dishwasher at home?” 5) “How many bathrooms (room with a bath/shower or both) are in your home?” 6) How many times did you and your family travel out of Wales for a holiday/vacation last year?” Scores for each item were summed to provide an overall indicator of student-level material affluence, with higher scores reflecting greater affluence.

#### Cigarette smoking

Students were asked; “How often do you smoke tobacco at present?” (response options: “every day”; “at least once a week, but not every day”; “less than once a week”; or “I do not smoke”). Those reporting smoking “every day” or “at least once a week, but not every day” were categorised as weekly smokers and assigned a value of 1, all other responses were coded 0.

#### Cannabis use

Students were asked; “Have you ever taken cannabis [in the last 30 days]?” (response options: “never”; “1–2 days”; “3–5 days”; “6–9 days”; “10–19 days”; “20–29 days”; or “30 days or more”). Any response indicating past month use of cannabis was classified as regular use and assigned a value of 1, all other responses were coded 0.

#### Truancy

School truancy was measured by the question; “In the past year, how many times did you truant from school for at least half a day (i.e. a morning or an afternoon)?” (response options: “never”; “once”; “two to four times”; “five or more times”). Students reporting any truancy were assigned a value of 1, all other responses were coded 0.

#### School exclusion

Students were asked; “Have you ever been excluded from school (suspended or expelled) because of your behaviour whilst at school?” (response options: “never”; “once”; or “more than once”). Responses indicating any prior exclusion were assigned a value of 1, all other responses were coded 0.

#### Mental wellbeing

The short Warwick-Edinburgh Mental Wellbeing Scale (SWEMWBS) [29] was used to measure student mental wellbeing. SWEMWBS comprises seven items relating to psychological functioning which asked about the following experiences over the last 2 weeks: a) I’ve been feeling optimistic about the future, b) I’ve been feeling useful, c) I’ve been feeling relaxed, d) I’ve been dealing with problems well, e) I’ve been thinking clearly, f) I’ve been feeling close to other people, g) I’ve been able to make up my own mind about things (response options: “none of the time”; “rarely”; “some of the time”; “often”; “all of the time”). Responses were assigned numerical scores (with higher within item scores indicating more positive mental wellbeing) and an overall score derived for each student based on the summation of individual item responses.

### Data linkage

Anonymised SHW survey data from non-HBSC schools were deposited within the Secure Anonymised Information Linkage (SAIL) Databank [30] and subsequently linked to a series of routine medical records in order to gather mental health related Read codes. The process by which datasets are anonymised and linked to routine data has previously been described in detail elsewhere [28]. Specifically, the following datasets were linked; primary care dataset (GPD), hospital inpatient dataset (PEDW), emergency department dataset (EDDS) and the hospital outpatient dataset (OPD). Data between 1st January 2009 and 31st August 2017 were used, providing full coverage of EDDS, PEDW, and OPD, while GPD covers 77%, i.e. 333/432, of all general practices in Wales. We defined GPD coverage as the cumulative amount of time an individual is registered with a GP providing data to SAIL. We identified diagnoses of mental health, self-harm, depression and anxiety, and contacts with a mental health specialty recorded in the GPD and PEDW. Mental health and self-harm diagnoses were also identified in the EDDS, and mental health specialty contacts in the OPD. These variables were defined by algorithms and lists of Read codes and ICD10 codes validated and elaborated in discussion with expert clinicians [6, 31].

### Data analysis

All statistical analyses were undertaken in Stata v.14. For the purpose of analysis, we restricted the sample to students in UK school years 7–11 (ages 11–16 years), excluding sixth form students in years 12 (16–17 years) and 13 (17–18 years). Student- and school-level characteristics were compared between data linkage pilot schools and the full sample of schools to examine the broader representativeness of pilot schools. Parental withdrawal rates were also calculated for linkage and non-linkage samples to assess any pre-existing bias in obtaining student consent, with school-level enrolment numbers for students in years 7–11 (obtained from the pupil level annual school census (PLASC) in Wales) used to construct denominator populations.

Multilevel logistic regression techniques were used to model consent (yes/no) to data linkage, with individual students nested within Welsh secondary schools to account for the hierarchical structure of the data. Missing data and responses of “I do not want to answer” (an additional response option to all questions included in the survey) were omitted from the analysis, except regarding gender where such responses were retained. The same analysis was used to model data linkage success (successful/unsuccessful) to ascertain the quality of identifiable data provided by the students consenting to linkage. For each ​​multilevel model, intraclass correlation ​coefficients (ICCs) ​were computed to denote the amount of dependency among observations within ​clusters using the *estat icc* command in Stata. Linkage success rate, including deterministic (i.e. an exact match using identifiers provided) and probabilistic matching with matching score ≥ 0.9 (i.e. a probabilistic match taking account of the probabilities of agreement and disagreement between a range of matching variables in the event that some identifiers are missing/inaccurate [8]) were quantified for the overall dataset and by individual characteristics (i.e. age, gender, socioeconomic status), drawing on comparisons between linked and unlinked records. Finally, we compared mental health diagnoses and health contacts between the linked SHRN cohort registered in the Welsh Demographic Service with the rest of the population in Wales of the same age, i.e. week of birth between 1st September 2000 and 31st August 2006 – students from years 7–11 (ages 11–16 years) in 2017. As an indication of GP data availability and comparability, we reported the mean (in years) and total (in person years) GPD coverage across individuals for both cohorts. Descriptive statistics were used to summarise proportions of students, including counts, percentages and 95% confidence intervals (CIs), estimated by Wilson score with continuity correction [32].

## Results

### Demographic characteristics of data linkage pilot schools

Data linkage pilot schools (*n* = 39) generally matched well with the full sample (*n* = 193 schools) regarding student socio-demographics and health risk behaviours (Table 1). Despite a few small but significant variations, overall, these figures demonstrate a good degree of comparability between samples.

**Table 1:** Comparison of student and school-level characteristics between samples

		School consented to data linkage question		
	Full SHW survey sample (*n* = 103,971; schools = 193)	Data linkage sub-sample (*n* = 18,956; schools = 39)	Data linkage control sample (*n* = 8374; schools = 22)^a^	Regression-based *P*-value (data linkage vs. non-data linkage sample)^b^
	n (%)	n (%)	n (%)	
***Student-level***				
Gender				
Male	50,452 (48.5)	9545 (50.4)	3788 (45.2)	0.078
Female	51,458 (49.5)	9064 (47.8)	4421 (52.8)	0.110
Did not want to answer	2061 (2.0)	347 (1.8)	165 (2.0)	0.283
School grade				
Year 7	22,634 (21.8)	3787 (20.0)	2064 (24.7)	0.005
Year 8	22,421 (21.6)	4122 (21.8)	1998 (23.9)	0.755
Year 9	22,208 (21.4)	4347 (22.9)	1628 (19.4)	0.023
Year 10	19,704 (19.0)	3500 (18.5)	1417 (16.9)	0.580
Year 11	17,004 (16.4)	3200 (16.9)	1267 (15.1)	0.552
Weekly cigarette smoker				
No	96,170 (96.6)	17,656 (96.9)	7745 (96.4)	–
Yes	3422 (3.4)	571 (3.1**)**	291 (3.6)	0.168
cannabis use				
No	94,156 (95.6)	17,799 (96.2)	7604 (95.5)	–
Yes	4305 (4.4)	699 (3.8)	357 (4.5)	0.061
Ever truant				
No	68,071 (73.9)	13,666 (75.4)	5457 (73.2)	–
Yes	23,989 (26.1)	4455 (24.6)	1995 (26.8)	0.046
Ever excluded				
No	86,614 (92.2)	17,216 (93.0)	6947 (91.1)	–
Yes	7370 (7.8)	1304 (7.0)	675 (8.9)	0.091
SWEMWBS (sd.)	21.955 (4.51)	22.094 (4.33)	21.867 (4.40)	0.048
FAS (sd.)	9.28 (2.34)	9.333 (2.32)	9.166 (2.34)	0.557
***School-level***				
Mean FAS (sd.)	9.309 (0.63)	9.353 (0.50)	9.192 (0.653)	0.595

Notes: SWEMWBS, Short Warwick-Edinburgh Mental Wellbeing Scale; FAS, Family Affluence Scale

^a^The data linkage control sample refers to the 22/64 schools which consented to data linkage, but which were not randomly assigned to the data linkage sample following the 2:1 randomization process (see Fig. 1)

^b^Variation in student socio-demographics and risk behaviours between samples were explored using binary and linear regression models (depending on the outcome variable) with adjustment for clustering by school. As the data linkage sample was nested within the full SHW survey sample, reported *p*-values were drawn from models comparing students attending data linkage (n = 39) versus non-data linkage (*n* = 154) schools

### Parental non-consent rates among linkage and non-linkage schools

In total, 137 schools (71%) participating in the 2017 SHW survey returned data on parental withdrawals; 28 (72%) data linkage schools and 109 (71%) non-linkage schools, the latter of which also included 14 (64%) data linkage control schools (schools that had consented to linkage but which had not been randomly assigned to the data linkage sample). Overall, 0.15% (95% confidence intervals [CIs]: 0.13, 0.17) of eligible students were withdrawn from the sample by parents. On average, data linkage schools reported a very marginally higher percentage of withdrawals (0.18%; 95% CIs: 0.14, 0.23) than non-linkage schools (0.14%; 95% CIs: 0.11, 0.16), and a marginally lower percentage compared to control schools (0.25%; 95% CIs: 0.19, 0.32); although these differences did not reach statistical significance, and opt-out remained sufficiently trivial as to be unlikely to introduce bias. Moreover, the removal of a prospective outlier school from each sample (responsible for 42.5, 25.7 and 58.8% of withdrawals in the data linkage, non-data linkage, and control samples, respectively) reduced these percentages to a comparable 0.11% (95% CIs: 0.10, 0.13), 0.10% (95% CIs: 0.09, 0.11), and 0.12% (95% CIs: 0.09, 0.14). Hence, there was no evidence that introduction of consent to link had an impact on parental consent rates.

### Demographic and behavioural predictors of young people’s consent to link in linkage pilot schools

Table 2 presents a breakdown of the socio-demographic and behavioural characteristics of students consenting to data linkage. Overall, 48.7% (*n* = 9232) of students within data linkage pilot schools consented to linkage and provided identifying information. A similar number of males (48.8%) and females (49.3%) provided consent to linkage, and consent rates were generally higher among younger-aged students: consent rates among year 7 students (ages 11–12) were up to 7% higher than those recorded for students in years 9 to 11.

**Table 2:** Modelled student and school level characteristics associated with assent to data linkage

		Data linkage sub-sample (*n* = 18,956)			
		Bivariate models		Multivariable model	
	Consented to data linkage, n/N (%)	OR (95% CIs)	Wald test	AOR (95% CIs)^c^	Wald test
***Student-level***					
Gender					
Male	4654/9545 (48.8)	1.00		1.00	
Female	4465/9064 (49.3)	1.01 [0.95, 1.07]		1.02 [0.95, 1.08]	
Did not state	113/347 (32.6)	**0.52 [0.41, 0.65]**	**X**^**2**^**(2) = 31.55**	**0.65 [0.50, 0.86]**	**X**^**2**^**(2) = 10.26**
School grade					
Year 7	1980/3787 (52.3)	1.00		1.00	
Year 8	2125/4122 (51.6)	0.97 [0.89, 1.07]		0.99 [0.90, 1.09]	
Year 9	1969/4347 (45.3)	**0.74 [0.68, 0.81]**		**0.76 [0.69, 0.83]**	
Year 10	1696/3500 (48.5)	**0.88 [0.80, 0.96]**		0.93 [0.84, 1.03]	
Year 11	1462/3200 (45.7)	**0.77 [0.70, 0.85]**	**X**^**2**^**(4) = 67.55**	**0.81 [0.73, 0.90]**	**X**^**2**^**(4) = 51.32**
Weekly smoker					
No	8794/17,656 (49.8)	1.00		1.00	
Yes	219/571 (38.4)	**0.66 [0.55, 0.78]**		1.02 [0.81, 1.30]	
Past month cannabis use					
No	8848/17,799 (49.7)	1.00		1.00	
Yes	279/699 (39.9)	**0.67 [0.57, 0.78]**		0.86 [0.70, 1.06]	
Ever truant					
No	6988/13,666 (51.1)	1.00		1.00	
Yes	2007/4455 (45.1)	**0.78 [0.73, 0.84]**		**0.91 [0.85, 0.99]**	
Ever excluded					
No	8633/17,216 (50.2)	1.00		1.00	
Yes	511/1304 (39.2)	**0.65 [0.58, 0.74]**		**0.82 [0.72, 0.94]**	
SWEMWBS (sd.)	22.415 (4.23)	**1.03 [1.03, 1.04]**		**1.02 [1.01, 1.03]**	
FAS (sd.)	9.457 (2.29)	**1.03 [1.02, 1.05]**		**1.02 [1.01, 1.04]**	
***School-level***					
Mean FAS (sd.)	9.397 (0.50)	**1.38 [1.04, 1.85]**		**1.36 [1.02, 1.83]**	
**ICC – constant only**	0.071				
**ICC – Student level**	0.071				
**ICC – Level 1 & 2 variables**	0.064				

Notes: ***P*** **< 0.05;** SWEMWBS, Short Warwick-Edinburgh Mental Wellbeing Scale; FAS, Family Affluence Scale, Intraclass correlation coefficient (ICC)

^c^Adjusted model: *n* = 17,256; schools = 39; observations per school: min = 121; max = 1003

Also reported in Table 2 are the results of the multilevel logistic regression models, exploring both bivariate (unadjusted) and multivariable (adjusted for age, gender, socioeconomic status) associations between student- and school-level characteristics and consent to data linkage. As shown, little observable difference was identified between the bivariate and multivariable models regarding the effects of socio-demographic factors on consent to linkage. Both models found no marked gender difference, although students who declined to state their gender were significantly less likely to consent to linkage relative to males. Students in school years 9 (ages 13–14) and 11 (ages 15–16) were also consistently less likely to consent to linkage relative to their younger contemporaries, with a trend toward lower consent with older age.

At the school level, aggregate FAS scores (estimated from mean student-level scores) were positively associated with consent to linkage with schools with higher scores (i.e. schools with more affluent overall intakes) having higher rates of linkage consent (adjusted OR [AOR] = 1.36; 95% CIs: 1.02, 1.83).

### Health risk behaviours and mental wellbeing

Students were less likely to consent to linkage if they reported adverse health behaviours including weekly cigarette smoking or regular use of cannabis, although neither association retained significance in the adjusted model, perhaps indicating that differences in unadjusted models were driven by the aforementioned tendency for lower consent among older adolescents. In comparison, students having ever truanted or having ever been excluded from school were consistently found to have a reduced likelihood of consenting to data linkage, even after controlling for other factors. By contrast, student-level FAS score (as an indicator of household socio-economic status) was positively related to a student’s likelihood of consenting to linkage. A similar association was also found for students reporting more positive mental wellbeing.

### Successful data linkage rates among students within linkage pilot schools who provided consent to link

Of those providing identifiable data and consent to link, 77.9% (*n* = 7192) of students provided a date of birth, of which 99.5% (*n* = 7158) were considered realistic (i.e. between 1991 and 2008). For postcode data, 61.4% (*n* = 5644) of students provided a complete postcode, of which 93.0% (*n* = 5248) were valid Welsh postcodes, while 2.4% (*n* = 225) provided partial postcode data (e.g. first four digits only) and 36.4% (*n* = 3363) provided no data. Approximately 55.9% (*n* = 5163) of students provided both a realistic date of birth and valid postcode. The provision of identifiable data enabled 69.8% (*n* = 6441) young people to be successfully linked. Of these, 66.4% (*n* = 4278) were deterministically linked.

### Demographic predictors of successful linkage among students providing consent

Table 3 presents a breakdown of the socio-demographic characteristics of linked students. Of those students who consented to linkage, a similar number of males (51.5%) and females (48.1%) were successfully linked. Successful linkage rates were generally higher among younger-aged students.

**Table 3:** Modelled student and school level characteristics associated with linkage success

	n (%)	Linked (%)	OR (95% CIs)	Wald test	AOR (95% CIs)	
**Student-level**						
Gender						
Males	4654 (50.4)	3315 (51.5)	1.00		1.00	
Female	4465 (48.4)	3098 (48.1)	**0.91 [0.82, 1.00]**		0.94 [0.85, 1.04]	
Did not want to answer	113 (1.2)	28 (0.4)	**0.11 [0.07, 0.18]**	**X**^**2**^**(2) = 31.55**	**0.19 [0.12, 0.32]**	**X**^**2**^**(2) = 40.33**
School grade						
Year 7	1980 (21.4)	1538 (23.9)	1.00		1.00	
Year 8	2125 (23.0)	1608 (25.0)	0.89 [0.76, 1.04]		0.89 [0.75, 1.05]	
Year 9	1969 (21.3)	1360 (21.1)	**0.63 [0.54, 0.73]**		**0.62 [0.53, 0.73]**	
Year 10	1696 (18.4)	1023 (15.9)	**0.43 [0.37, 0.5]**		**0.43 [0.36, 0.50]**	
Year 11	1462 (15.8)	912 (14.2)	**0.41 [0.35, 0.48]**	**X**^**2**^**(4) = 212.34**	**0.39 [0.33, 0.47]**	**X**^**2**^**(4) = 189.57**
SWEMWBS (sd.)	22.449 (4.24)	22.651 (4.19)	**1.04 [1.03, 1.05]**		**1.03 [1.01, 1.04]**	
FAS (sd.)	9.484 (2.27)	9.504 (2.24)	1.00 [0.98, 1.03]		1.00 [0.97, 1.02]	
**School-level**						
Mean FAS (sd.)	9.484 (0.51)	9.499 (0.52)	1.11 [0.75, 1.63]		1.15 [0.76, 1.76]	
**ICC – constant only**	**0.119**					
**ICC – Student level**	**0.128**					
**ICC – Level 1 & 2 variables**	**0.126**					

*n* = 9232 (adjusted model *n* = 8401); schools = 39; observations per school: min = 41; max = 817; *P < 0.05* Intraclass correlation coefficient (ICC)

Also reported in Table 3 are the results of the multilevel logistic regression models, exploring associations between student- and school- level characteristics and linkage success. Little observable difference was identified between the bivariate and multivariable models regarding associations of socio-demographic factors with consent to linkage. Again, no marked gender difference was observed, although as expected students who declined to state their gender were significantly less likely to be successfully linked due to our linkage methodology. Chances of successful linkage decreased steadily with older ages. SWEMWBS scores were weakly associated with linkage success, with higher scores slightly increasing the odds of successful linkage. Both at student- and school-level, FAS scores were not associated with linkage success. For assent to linkage, the ICC of 0.071 within both the null model and the model adjusted for student level (compositional) variables only indicated approximately 7% of variance at the school level. This was only marginally reduced after adjustment for school-level (contextual) variables. For linkage success, a somewhat higher proportion of variance at the school-level was observed (12% in null models and 13% in adjusted models).

### Mental health status of linked cohort compared to the general population

Of the 6441 linked students, 6419 (99.7%) were found in the Welsh Demographic Service and compared with the 232,596 additional young people with week of birth between 1st September 2000 and 31st August 2006. In total, 78.3% (5029/6419) of the linked cohort had GPD data available between 1st January 2009 and 31st August 2017, totalling 37,719.69 person years of GPD data, each contributing an average of 6.19 years. Similarly, 75.4% (175,448/232,596) of the same age general population had GPD available between 1st January 2009 and 31st August 2017, totalling 1,287,625.64 person years of GPD data, each contributing an average of 5.54 years.

Table 4 displays the proportion of mental health diagnoses and related specialty contacts among those in the linked cohort and among similar aged peers in the general population of Wales. As shown, for each outcome, students in the linked cohort had a lower proportion of both diagnoses (5.8% vs. 8.8%, *p* < 0.001) and overall number of specialty contacts (5.2% vs. 7.7%, p < 0.001). Findings were consistent when looking at specific types of diagnoses, for example proportions of depression and anxiety, and self-harm.

**Table 4:** Routine records of mental health diagnoses and specialty contacts among linked cohort versus Welsh population

	Linked cohort	Welsh population (aged 11–16 yrs)	
	n (%) [95% CIs]		
Any mental health diagnoses	373 (5.8%) [5.26, 6.42]	20,551 (8.8%) [8.72, 8.95]	
Contact with a mental health speciality	334 (5.2%) [4.68, 5.78]	17,994 (7.7%) [7.63, 7.85]	
Depression & anxiety diagnoses	125 (2.0%) [1.63, 2.32]	8088 (3.5%) [3.40, 3.55]	
Self-harm	36 (0.6%) [0.40, 0.78]	3311 (1.4%) [1.38, 1.42]	

## Discussion

This study demonstrates that while the introduction of consent to data linkage within a representative secondary-school survey may not be of detriment to survey completion rates, sources of bias are evident at both the student consent and data linkage stages.

### Parental consent

Passive parental consent was one of three levels of consent required to participate in the survey, alongside both school and student levels. In contrast to active (i.e. opt-in) consent, passive consent was preferred to encourage both a greater response rate and a more representative socio-demographic sample [20]. We found little evidence that requesting permission to link data reduced parental consent rates, with marginal withdrawal differences between data linkage and non-data linkage schools, diminishing further following the exclusion of prospective outlier schools (i.e. one school was responsible for 42.5% of all parental opt-outs in the data linkage sample). Thus, including an option for data linkage within a school-based survey may not substantially alter overall participation rates.

### Individual- and school-level predictors of consent among students not withdrawn by parents

Approximately one-in-two young people aged 11–16 years consented to data linkage within the present study; a rate comparable to that of adult populations (typically ranging between 34 and 86%) [33, 34]. Regarding demographic predictors of consent, student gender appeared to have little discernible effect on decision to consent, a contrasting finding to that of Ullemar and colleagues, who explored provision of consent to accessing health records among a Swedish adolescent twin cohort [35]. They found that males were 25% less likely to consent compared to females. In the present study, higher consent rates were however observed among younger-aged students and students reporting a higher family affluence score, with results persisting following adjustment for other factors.

Further bias was also apparent when considering student’s self-reported behaviours, with lower consent rates observed among students who reported risk-related behaviours such as truancy and school exclusion. Reports of smoking and cannabis use were identified as significant predictors of lower consent in bivariate analyses only, potentially signifying a confounding association with age, given the higher age of smokers and the greater likelihood of non-consent among older students. This is in contrast to Baghal [36] who found no observable difference in adolescent smoking rates when seeking consent to link survey and administrative records. Additionally, we found that students who reported more positive mental wellbeing were more likely to provide consent for data linkage. Overall, these findings are generally consistent with previous studies exploring sample bias within self-report surveys that have tended to observe lower levels of participation among young people who engage in more risk-related behaviours [37, 38]. As such, our findings have clear implications for hybrid cohort studies that rely on linked data, such as epidemiological and wider evaluative studies, particularly concerning the external validity of conclusions drawn from such data [26] given the potential under-representation of certain cohorts within the linked sample.

School-level influences can also impact upon student behaviour: students attending schools with higher levels of absenteeism, for example, have a greater tendency to engage in risk-taking behaviours [37]. In the current study, there was substantial clustering in consent to linkage at the school level, while school level affluence was positively associated with consent to linkage, independent of student affluence and other factors. This demonstrates the potential for an additional layer of bias with a young person’s decision to consent to linkage also dependent upon characteristics relating to their school environment. Substantial school level variance in linkage consent might reflect unmeasured compositional differences in characteristics of pupils between school, or might also reflect differences in the context of schools and the ways in which the survey was completed in schools. School level variance in linkage success may reflect these differences, as well as additional clustering in the coverage and quality of local administrative data to which pupils’ surveys were linked. Future work will seek to explore wider measures of school-level policies to ascertain how these may impact upon parent and pupil decision making.

### Data linkage rates among consenting students

Alongside the decision to consent, data linkage depends on the accurate provision of identifiable data required to successfully facilitate linkage. Hence the quality of data provided can impact upon the precision of subsequent analyses [39], with inaccurate reporting of personal details leading to unmatched (records belonging to the same individual are not linked) or falsely matched (records of two different individuals are erroneously linked) data [40]. In this study, around two-thirds of students provided the required identifiable data, whereas the remaining students provided either incomplete data or no data at all for some or all the requested fields (i.e. full name, date of birth, and UK post code).

We observed that successful linkage decreased with age but was unaffected by student gender, implying that younger students can correctly provide details on home address. SWEMWBS scores were weakly associated with linkage success, with higher scores slightly increasing the odds of successful linkage. Furthermore, compared to peers in the general population of Wales, we found that students with successful linkage were less likely to have a mental health diagnosis, a contact with specialist mental health services or self-harm. As such, this study highlights the need to increase levels of accurate reporting to gain meaningful insights, with an exploration into the possibilities for automated linkage and or refinement of processes for the reporting of identifiable information.

### Strengths and limitations

To the best of our knowledge, this is the first UK-based study to examine potential biases associated with the introduction of consent to data linkage and subsequent linkage success within a national survey of secondary school-aged children. A clear strength of this pilot is the socio-demographic representativeness of students sampled in data linkage pilot schools relative to the whole sample. Given the use of an embedded pilot design, encompassing around 18% of Welsh secondary schools, such comparability was not guaranteed, and provides greater confidence in the wider generalisability of our findings. Moreover, we have shown that parental opt-out procedures are viable when seeking consent for data linkage within a national student survey, revealing minimal effects on survey completion rates and corresponding student reports. There were some limitations, however: we cannot rule out the possibility that requesting identifiable data may have led to under-reporting of certain behaviours given the sensitivity of some topics included in the survey (e.g. substance use). Although comparisons with previous versions of the survey [41, 42] suggest that this was not the case. Additionally, as demographic and behavioural characteristics were only collected from students, we were unable to draw any inferences on the factors determining parental consent – factors that will directly impact upon student participation rates considering parental consent precedes student consent.

Regarding our analysis of routine data, because GPD coverage was not complete for the considered study period, our results may be underestimations and are therefore conservative. At the same time, the fact that both cohorts (linked and Welsh population) had comparable GPD coverage greatly ameliorates any possible bias in the ORs as a result of data coverage, supporting our conclusions derived from this comparative analysis. Lastly, the rate of consent to linkage halved our initial study population. Therefore, in addition to the reported sources of biases outlined within this study, future analyses of this dataset must also consider study power.

### Implications

This study has explored the potential for linking young people’s survey data to anonymised routine datasets. The ability to create an electronic cohort is an increasingly attractive option offering cost benefits, opportunities for longitudinal follow-up with reduced participant burden and avenues for a detailed and timely evidence-base. That said, it is forecast that the demand for administrative data linkage in surveys is likely to increase [43]. It is therefore vital that future research explores the reasons for parental and child non-consent alongside potential facilitators in order to maximise the accuracy of data used for linkage, and subsequently the representation of future longitudinal cohorts. One avenue for overcoming the potential biases within the linked dataset, is for future analyses to use the larger population survey data as auxiliary data from which survey weights can be calculated.

## Conclusion

Data linkage provides a powerful tool for longitudinal research, but our study highlights the importance of taking into consideration potential sources of bias. We found that routine administrative data successfully linked to student survey data may not be representative of the total student population, with biases related to i) school-level characteristics, ii) student characteristics, and iii) the accuracy of the provision of identifiable data.

Exploring reasons for parental- and child opt-out, along with approaches to maximise the accuracy of reporting identifiable data will help improve the reliability and validity of analyses based on linkage of routine data to survey data.

## Data Availability

The data that support the findings of this study are available from DECIPHer but restrictions apply to the availability of these data, which were used under license for the current study, and so are not publicly available. Data are however available from the authors upon reasonable request and with permission of DECIPHer.

## Abbreviations

AOR: Adjusted Odds Ratio
EDDS: Emergency Department Dataset
FAS: Family Affluence Scale
GPD: General Practice Database
HBSC: Health Behaviour in School-aged Children
ICC: Intraclass correlation coefficient
NHS: National Health Service
OR: Odds Ratio
OPD: Outpatient Dataset
PEDW: Patient Episode Database Wales
PLASC: Pupil Level Annual School Census
SHRN: School Health Research Network
SWEMWBS: Short Warwick-Edinburgh Mental Wellbeing Scale
SHW: Student Health and Wellbeing
UK: United Kingdom
